# Building the capacity of family day care educators to promote children's social and emotional wellbeing: an exploratory cluster randomised controlled trial

**DOI:** 10.1186/1471-2458-11-842

**Published:** 2011-11-03

**Authors:** Elise Davis, Lara Williamson, Andrew Mackinnon, Kay Cook, Elizabeth Waters, Helen Herrman, Margaret Sims, Cathrine Mihalopoulos, Linda Harrison, Bernard Marshall

**Affiliations:** 1 Jack Brockhoff Child Health and Wellbeing Program, McCaughey Centre, University of Melbourne, Level 5, 207 Bouverie Street, Carlton, Victoria, 3152, Australia; 2 ORYGEN Youth Health Research Centre, Locked Bag 10 Parkville, Victoria 3052 Australia; 3 Deakin University, 221 Burwood Highway, Burwood, Victoria, 3123, Australia; 4 University of New England, Elm Avenue, Armidale, New South Wales, 2051, Australia; 5 Charles Sturt University, Mt Panorama Avenue, Bathurst, NSW, 2795, Australia

## Abstract

**Background:**

Childhood mental health problems are highly prevalent, experienced by one in five children living in socioeconomically disadvantaged families. Although childcare settings, including family day care are ideal to promote children's social and emotional wellbeing at a population level in a sustainable way, family day care educators receive limited training in promoting children's mental health. This study is an exploratory wait-list control cluster randomised controlled trial to test the appropriateness, acceptability, cost, and effectiveness of "Thrive," an intervention program to build the capacity of family day care educators to promote children's social and emotional wellbeing. Thrive aims to increase educators' knowledge, confidence and skills in promoting children's social and emotional wellbeing.

**Methods/Design:**

This study involves one family day care organisation based in a low socioeconomic area of Melbourne. All family day care educators (term used for registered carers who provide care for children for financial reimbursement in the carers own home) are eligible to participate in the study. The clusters for randomisation will be the fieldworkers (n = 5) who each supervise 10-15 educators. The intervention group (field workers and educators) will participate in a variety of intervention activities over 12 months, including workshops; activity exchanges with other educators; and focused discussion about children's social and emotional wellbeing during field worker visits. The control group will continue with their normal work practice. The intervention will be delivered to the intervention group and then to the control group after a time delay of 15 months post intervention commencement. A baseline survey will be conducted with all consenting educators and field workers (n = ~70) assessing outcomes at the cluster and individual level. The survey will also be administered at one month, six months and 12 months post-intervention commencement. The survey consists of questions measuring perceived levels of knowledge, confidence and skills in promoting children's social and emotional wellbeing. As much of this intervention will be delivered by field workers, field worker-family day care educator relationships are key to its success and thus supervisor support will also be measured. All educators will also have an in-home quality of care assessment at baseline, one month, six months and 12 months post-intervention commencement. Process evaluation will occur at one month, six months and 12 months post-intervention commencement. Information regarding intervention fidelity and economics will also be assessed in the survey.

**Discussion:**

A capacity building intervention in child mental health promotion for family day care is an essential contribution to research, policy and practice. This initiative is the first internationally, and essential in building an evidence base of interventions in this extremely policy-timely setting.

**Trial Registration number:**

343312

## Background

Early mental health promotion is important for prevention of mental disorders and for mental health status throughout life. The period from conception to school age is critical to neural wiring and brain development [1]. Mental health problems have significant and long-term personal, social and economic implications for the individual, their family and the wider community. Childhood mental health problems are highly prevalent, experienced by one in seven Australian children aged between 4-17 years [2]. There are marked inequalities in the distribution of mental health problems, with the rate increasing to one in five children for those living in low-income or single parent families [2]. Mental health problems are also apparent in young children [3,4]. For example, a recent study reporting population data from the Longitudinal Study of Australian Children found that 11.5% of children aged 4-5 years had scores indicating abnormal or concerning mental health [5].

Child mental health is more than the absence of mental illness; it is "the achievement of expected developmental cognitive, social, and emotional milestones and by secure attachments, satisfying social relationships, and effective coping skills" (p.123) [6]. Positive mental health, though variously defined, includes emotion (affect/feeling), cognition (perception, thinking, reasoning), social functioning (relations with others and society), and a sense of meaning in life [7]. To promote positive mental health, infants and children require good maternal health, adequate nutrition, secure attachments with caregivers, and caregivers who are knowledgeable, skilled and competent with access to support services and networks [8].

Childcare settings are ideal to promote children's social and emotional wellbeing and to identify early mental health problems in the population in a sustainable way, given the large number of children who attend. In Australia, use of childcare has increased in recent years, with 35.2% of all children aged 0-4 years accessing some form of formal childcare in 2005 compared to 23.6% in 1996 [9].

Family day care (FDC), where registered educators provide formal paid care in their own homes for other people's children under the management of a local coordination scheme, is a popular form of childcare in Australia. It is utilised by 20.5% of all parents of children aged 0-4 years who accessed some form of formal care in 2004 [9,10]. Recent changes in Victorian childcare regulations now require all educators to have attained or to be working towards gaining a formal childcare qualification. The minimum level of attainment is a Certificate III in Children's Services which requires around 12 months study. Upon its completion the educator can then undertake an additional year of study to qualify for the Diploma in Children's Services. Recent surveys illustrate an increase in FDC educator qualifications, from 25% holding a Certificate III in 2004 [8] to over 50% in 2010 [11]. This is significant given that both educator education and training are better predictors of childcare quality than educator age and work experience [12]. Despite this, FDC educators do not study mental health promotion in the curriculum of the Certificate III or the Diploma in Children's Services nor is this type of training common in professional development programs.

In the absence of any international research examining the knowledge, skills and competencies of FDC educators in the area of child mental health promotion, our team conducted a qualitative study which highlighted several challenges faced by educators [13]. Educators had difficulty identifying the causes and early signs of mental health problems for children. The strategies they used to promote children's mental health were informal and dependent on the educator's individual skills. They had difficulty identifying mental health promoting policies, and connecting families with community health services. Common barriers to mental health promotion include financial resources, a lack of knowledge about child mental health, and a fear of discussing mental health concerns with parents. Educators also requested training in child mental health and communication with parents.

To date, only one intervention program has been developed to promote Australian childcare workers' (including family day care educators') knowledge of mental health problems. The Healthy Start Program [14] involved training for child educators in risk and protective factors of mental health, and communication with parents around child mental health issues. The intervention program was associated with an increase in educators' confidence, skills and knowledge about promoting children's mental health at six months; however these gains were not retained when educators were re-tested at 12 months. Although evidence suggests that training improves the competencies of child educators (professional attitude, knowledge and skills) [12] and that family day care educators who participate in training offer higher quality care than providers who do not participate [15], for training to be effective and long lasting, it has to be part of a strategy that addresses organisational policies, procedures, resources, standards of practice and supervision [16], consistent with a capacity building approach.

Capacity building involves actions aimed at strengthening the skills and capabilities of individuals, organisations, systems and communities [17]. Capacity building strategies for mental health promotion have been developed for school settings, including mental health promoting policies, curricula, and systems across the whole sector, building the skills and knowledge of teachers, as well as strengthening links with other organizations and groups within the community [18,19]. A new program, KidsMatter Early Childhood, an extension of MindMatters (secondary school) and KidsMatter (primary school) has recently been developed to fill this gap and has been trialled. KidsMatter Early Childhood aims to: "improve the mental health and wellbeing of children from birth to school age; reduce mental health problems among children and; achieve greater support for children experiencing mental health difficulties and their families" (http://www.kidsmatter.edu.au). Due to logistical challenges, FDC was not included in the implementation of this program. In the absence of any programs that aim to build the capacity of family day educators in order to promote children's social and emotional wellbeing, a new program has been developed ("Thrive: Promoting Children's Social and Emotional Wellbeing in Family Day Care").

### Development of Thrive

The initial stages of the project comprised telephone interviews and focus groups with FDC educators. Their responses guided the development of the intervention program. Telephone interviews were completed with 50 educators to help gain insight into their knowledge and confidence in promoting children's social and emotional wellbeing. This information provided useful insights and allowed educators to be categorised into different 'stages of change' [20] around their willingness to promote children's social and emotional wellbeing. The Stages of Change model has been widely used in health promotion programs and describes the stages an individual or organisation moves through from before they contemplate changing, to contemplating change, taking action, maintaining the new practice and also relapsing. These categories were used to assemble focus groups bringing together educators who were at similar stages of willingness to actively promote children's social and emotional wellbeing in their FDC practice. Four focus groups were conducted (three with educators in the action/maintenance phase, and one with fieldworkers) and five individual interviews with educators in the 'relapse', 'pre-contemplation' and 'preparation' stages. In addition, system perspectives were gained through six key informant interviews with representatives from peak bodies, training organisations, scheme sponsor management and government.

The Thrive program includes several activities for field workers and FDC educators. These activities include workshops for field workers (N = 4) and FDC educators (N = 3), activity exchanges with other educators and focused discussion on social and emotional wellbeing during field worker visits. The intervention group will also receive resources associated with the workshops on promoting children's social and emotional wellbeing. A cluster randomised controlled trial design is being used because the effectiveness of some of the intervention activities (i.e. activity exchange and field worker visits) is dependent on the field worker.

#### Aims

This study is the first stage in evaluating the appropriateness, acceptability, cost and effectiveness of an intervention program to build the capacity of FDC educators to promote children's social and emotional wellbeing. It is hypothesised that the intervention program will:

a) increase FDC educators' knowledge, confidence and skills in promoting children's social and emotional wellbeing

b) increase field workers' knowledge and confidence in promoting children's social and emotional wellbeing

c) build the capacity of the FDC organisation (as measured by workforce development, resource allocation and leadership).

This is the first intervention program designed to build the capacity of FDC educators to promote children's social and emotional wellbeing internationally.

## Methods/Design

### Study Design

Approval for the trial has been obtained from The University of Melbourne Human Research Ethics Committee (HREC 1136446). This study uses a wait-list control cluster randomised controlled trial to test the appropriateness, acceptability, feasibility, costs and effectiveness of the Thrive intervention program. The intervention program is being conducted with one FDC scheme based in a low socioeconomic area of Melbourne. A low socioeconomic area was selected because the prevalence of child mental health problems is higher in poorer areas [2].

**Figure 1:**
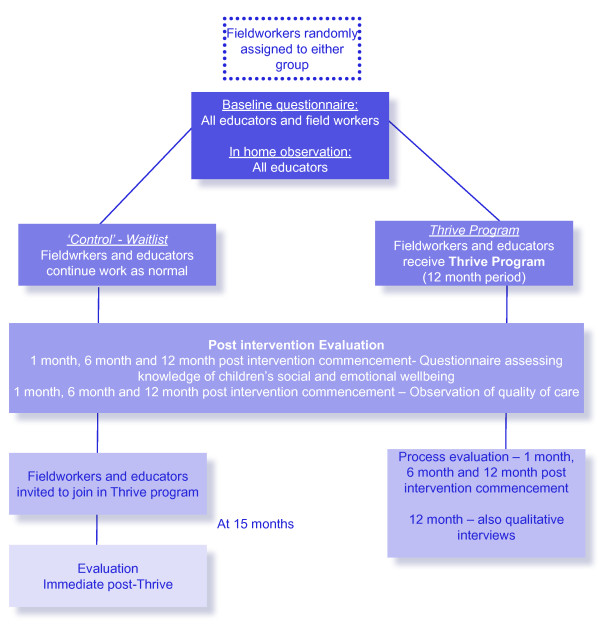
**Thrive randomised control trial flow chart**.

Figure 1 summarises the study design and timelines. All FDC educators and field workers within the scheme are eligible to receive the intervention program. The clusters for randomisation will be the field workers (n = 5) who each supervise 10-15 educators. Randomisation will be conducted in accordance with ICH Guideline E9 [21] by CI Mackinnon, who is independent of the administration of the intervention.

The intervention group will receive the intervention over a 12-month period, during which time the control group will continue standard practice. The intervention program will be delivered to the intervention group and then to the control group after a time delay of 15 months post intervention commencement. The Thrive intervention program includes several activities for field workers and FDC educators. These activities include workshops for field workers (N = 4) and family day care educators (N = 3), activity exchanges with other educators coordinated by their fieldworker and focused discussion on social and emotional wellbeing during field worker visits. The intervention group will also receive resources associated with the workshops on promoting children's social and emotional wellbeing.

All FDC educators will be informed that the intervention will roll out in a two-stage process. Fieldworkers will not be blinded as to which arm they are involved in with educators but will be made aware of the blinding process and its necessity. The researchers involved in data collection will be blinded as to which intervention educators are receiving.

### Participants and Recruitment

A cover letter, plain language statement and consent form describing the Thrive intervention and evaluation will be mailed to all educators, fieldworkers and management. An administrative assistant from the FDC scheme will then telephone educators to determine if they are interested in participating in the study. If so, administrators will request consent from the subjects to pass on their contact details to researchers. The researchers will then call the subjects to explain the study again and ask them if they would like to participate. If they are happy to participate, the researchers will organise a time for the baseline assessment.

#### Data Collection

A baseline survey will be conducted with all consenting educators and field workers (n = ~70). A survey will be administered at one month, six months and 12 months post-intervention commencement. All educators will also have an in-home quality-of-care assessment at baseline, one month, six months and 12 months post-intervention commencement.

The initial survey will collect demographics information including: educator age; preferred language; reason for becoming an FDC educator; hours attending professional development annually; qualifications (completed and in progress); number of years working in family day care; and number and characteristics of children in care.

### Primary Outcome

Conceptually, the primary outcome is knowledge about children's social and emotional wellbeing. In the absence of any existing relevant items, a set of items have been developed specifically for the trial. These measure this construct, including general questions of 'How would you rate your knowledge about children's social and emotional wellbeing?' and 'How would you rate your knowledge of who to contact and what to do if you are worried about the social and emotional wellbeing of a child in your care' (Scale 0-10 with 0 = almost no knowledge, 10 = very knowledgeable). Open-ended questions about risk and protective factors for good/poor mental health and early signs of poor mental health have also been developed.

### Secondary Outcomes

Secondary outcomes include confidence and skills in promoting children's social and emotional wellbeing, and organisational capacity building. In the absence of pre-existing relevant survey items on confidence in promoting children's social and emotional wellbeing, new items have been developed to measure this construct. Confidence is measured by four self-report questions 'Overall how confident are you in your ability to promote children's social and emotional wellbeing', 'How confident are you in your ability to identify children's social and emotional problems?', How confident are you in talking with parents about promoting their children's social and emotional wellbeing?' and 'How confident are you in talking with parents about potential problems with their children's social and emotional wellbeing?' (Scale 0-10 with 0 = not confident, 10 = very confident).

Skills will be measured in the survey by asking about the participant's daily activities (scale items from the Longitudinal Study of Australian Children) and by asking for a description of their FDC practice 'Please tell us about the ways you promote the social and emotional wellbeing of children in your care?' Skills in promoting social and emotional wellbeing are also assessed by objectively measuring quality of care (see In Home Assessments section). Organisational mental health promotion will be assessed using a mental health promotion audit at baseline and at six months and 12-months post intervention. This audit, which assesses organisational structure, workforce development, resource allocation and leadership will be completed in a one-on-one interview with the scheme manager. The interview is expected to take around 30-45 minutes. Example items include "How is promoting children's social and emotional wellbeing incorporated in to your fieldwork and management roles? Have fieldworkers, educators undertaken any professional development specific to promoting children's social and emotional wellbeing? How available and easy to access is information about promoting children's social and emotional wellbeing?"

### Mediation of outcomes

Supervisory support (field worker support of educators or managerial support of fieldworkers) will be assessed as it is a potential mediator and may influence the effectiveness of the intervention. The supervisory support subscale of the Multidimensional Support Scale [22] was used in the survey. Response options were adapted by rewording and expanding the number of response options from three options (not enough, they're okay, very satisfactory) to five (never, rarely, sometimes, often, always) to allow a greater range of responses and increased interpretability. Support is assessed by inquiring about how much a supervisor listens and understands the worker, how often they help the worker in practical ways or give clear and useful advice. The scale also queries whether the worker can use the supervisor's behaviour as an example to deal with problems.

### In home assessments

Quality of the FDC environment will be assessed through observations carried out at baseline, one month, six months and 12 months post-intervention by a trained researcher using the Family Child Care Environment Rating Scale Revised Edition (FCCERS-R) and the Caregiver Interaction Scale (CIS) [23,24]. The FCCERS-R has 38 items in 7 subscales. Four subscales (24 items) will be used in this study specific to mental health: personal care routines; listening and talking; activities; and interactions. Each item is scored from 1 (inadequate) to 7 (excellent). The FCCERS-R has high inter-observer reliability (0.83-0.90) and moderate to high internal consistency for the subscales (0.70-0.93).

The CIS has 26 items divided into four subscales that measure sensitivity, harshness, detachment and permissiveness of caregivers in the childhood care/education environment. Items are scored from 1 (not at all true) to 4 (very much true). It has a moderate to high inter-observer reliability (0.75-0.97) and high internal subscale consistency (0.81-0.91). In this study, we expect to see improvements in the four subscales of the FCCERS-R and in the CIS, reductions in harshness and detachment and improvements in the sensitivity subscales. The total time commitment for the observation is 2 hours.

### Process evaluation

Process evaluation will occur one month, six months and 12 months post-intervention commencement, when field workers and educators in the intervention group will complete a process evaluation survey to assess the quality of the intervention, intervention fidelity and economic viability. In addition, 12 months post-intervention commencement one focus group will be conducted with a sample of approximately 8-10 educators in the intervention group and another with all of the field workers (n = 5).

### Costing of the Intervention

Questionnaires regarding the cost associated with the intervention at the level of educator, coordination staff and FDC agency have been developed which will be administered at the same time as the survey. Costs assessed include: time spent travelling to, preparing for or participating in intervention activities; transport to the intervention (petrol, parking); loss of income; purchase of resources associated with promoting children's mental health; devotion of coordination staff time to the intervention; and providing a variety of intervention activities (venue hire, catering, facilitator etc). As this study is not an effectiveness trial only costs associated with the intervention will be assessed rather than a comprehensive evaluation of all costs associated with the intervention.

### Data Analysis

Primary analyses will be undertaken on an intent-to-treat basis, including all participants randomized regardless of the extent of participation in the intervention or withdrawal from the study. Mixed-model repeated measures analyses will be used because of the ability of this approach accommodate clustering effects, appropriately model the relationship between measures over time and to include participants with missing data [25,26]. Planned contrasts will test hypotheses address the effectiveness of the program compared to control at post-test, and six months and 12 months follow-up. Exploratory analyses of clustering (i.e. field worker) effects will be undertaken including estimation of the intra class correlation (ICC). Of particular interest are the effects at post-test after field workers have delivered the intervention. While the number of field workers is small, it is hoped to enhance precision of estimation by using the pre-, post-intervention data from control participants from the phase of the trial where they receive the intervention.

### Sample Size

Sample size is constrained to 35 per arm by the size of the organization hosting the trial. Nevertheless, under moderate assumptions, the study may have power to detect moderate size effects in its own right. Given the nature of the intervention and outcomes of comparable studies [27] such an effect is achievable and lies at the lower end of interest. Allowing for an ICC of 0.03 and average cluster size of 14 yields a design effect of 1.72 implying an effective sample size of 20 participants per arm. Assuming a correlation of 0.7 between pre- and post-test scores, the study will maintain 80% power to detect mean difference in change scores between groups of 0.7 standard deviations. Compared to trials of individual therapies, drop out in this study is expected to be restricted to normal worker turnover that is low and likely to be limited to the 12 month follow-up. It is acknowledged that precision in detecting between field worker differences and estimating ICCs will not be high. This is consistent with the exploratory nature of this study.

## Discussion

Childhood mental health problems are highly prevalent. The FDC system is an ideal sector to promote child mental health. This project aims to conduct an exploratory wait-list control cluster trial of a mental health promotion intervention for children in FDC. It will explore the feasibility, costs and effectiveness of an intervention that builds the capacity of FDC settings to promote children's mental health. This initiative is the first internationally, and is essential to build an evidence base of interventions in this extremely policy-timely setting.

Rarely is solution-oriented intervention research designed and conducted with a rigorous methodology in a community of extreme disadvantage. One of the motivations for this research is that it might serve as a model for the FDC and childcare sectors. With a strong evidence base, the THRIVE program may be implemented widely within the sector nationally, thus addressing a key area of children's health inequalities - mental health. Initial results of this study will become available in 2012.

## Abbreviations

FDC: Family Day Care; FCCERS-R: Family Child Care Environment Rating Scale Revised Edition; CIS: Caregiver Interaction Scale; ICC: Interclass Correlation

## Competing interests

The authors declare that they have no competing interests.

## Authors' contributions

Led by ED, all authors contributed to the grant application. ED and LW collected data for the qualitative studies underpinning the development of Thrive. All authors contributed to the analysis of the qualitative data and development of the Thrive program. All authors contributed to the study design. All authors read and approved the final manuscript.

## Authors' information

Elise Davis is a Senior Research Fellow at the University of Melbourne. Her research interests include child mental health promotion in the early childhood period. Lara Williamson has a Masters in Public Health and is a Research Fellow and PhD scholar at the University of Melbourne. Her research interests include mental health promotion, and women's and children's health. Andrew Mackinnon is a Professorial Fellow in the Centre for Youth Mental Health at The University of Melbourne and heads the Statistics Unit at Orygen Youth Health Research Centre. He has been involved in a range of mental health related epidemiological surveys as well as in designing, conducting and analysing psychiatric trials. Kay Cook is a Senior Research Fellow at Deakin University. Her research interests and expertise lie in qualitative research methods, the experience and processes of marginalisation, and how social policies shape the identity, experiences and outcomes of marginalised groups. Lisa Smyth is the Manager of Windermere Family Day Care Scheme and an executive member of Family Day Care Victoria. Her work involves building the capacity of family day care. Elizabeth Waters has a DPhil and an interest in child public health and child health inequalities, and is working on a program of research around mental health promotion for carers and young children. Helen Herrman is Professor of Psychiatry at the Centre for Youth Mental Health, University of Melbourne, and Director of the World Health Organization Collaborating Centre for Mental Health in Melbourne, Victoria, Australia. Margaret Sims is Professor of early childhood at the University of New England. Her research focuses on quality community based services for young children and families (child care, family support, integrated service delivery). Linda J. Harrison is Associate Professor of Early Childhood Education at Charles Sturt University and a member of the Consortium Advisory Group for the Longitudinal Study of Australian Children. Her research centres on quality practices in child care, children's socio-emotional development and wellbeing, and caregiver-child attachment relationships. Bernie Marshall is the Associate Dean (Teaching and Learning) of the Faculty of Health, Deakin University. He has over 20 years experience in research and consultancy related to health promoting schools. Cathrine Mihalopoulos is a senior health economist at Deakin Health Economics, Deakin University. Her primary research interests include the economic evaluation of mental programs and interventions.

## Pre-publication history

The pre-publication history for this paper can be accessed here:

http://www.biomedcentral.com/1471-2458/11/842/prepub
